# Advances of clinical trials on compound Danshen dripping pills for stable angina pectoris: A perspective

**DOI:** 10.1097/MD.0000000000042175

**Published:** 2025-04-18

**Authors:** Xiao-feng Liu, Xiao-xiao Zhu

**Affiliations:** a Department of Chinese Medicine Pharmacy, Shengzhou People’s Hospital (Shengzhou Branch of the First Affiliated Hospital of Zhejiang University School of Medicine), Shengzhou, China; b Department of Pharmacy, Shengzhou People’s Hospital (Shengzhou Branch of the First Affiliated Hospital of Zhejiang University School of Medicine), Shengzhou, China.

**Keywords:** angina pectoris, anti-inflammatory, compound Danshen dripping pills, mechanistic study, randomized controlled trial, stable angina pectoris, vasodilatory

## Abstract

Stable angina pectoris (SAP) is a prevalent manifestation of ischemic heart disease, characterized by chest pain due to myocardial ischemia. Although standard pharmacological treatments such as nitrates, beta-blockers, and calcium channel blockers are commonly prescribed, their long-term efficacy is often limited by side effects, prompting interest in alternative therapies. Compound Danshen droplet Pills (CDDP), a traditional Chinese medicine formulation comprising Salvia miltiorrhiza, Panax notoginseng, and Borneol, has been widely used in China for its cardiovascular benefits, including improving circulation and alleviating angina symptoms. Despite its clinical use, systematic evaluations of CDDP’s efficacy in SAP management are limited. This review summarizes the clinical evidence on CDDP for SAP treatment by analyzing randomized controlled trials (RCTs). A comprehensive search of the China National Knowledge Infrastructure and PubMed databases was conducted, covering studies up to July 31, 2024, using terms such as “compound Danshen droplet pills” and “stable angina pectoris.” Only peer-reviewed RCTs focusing on CDDP for SAP, published in English or Chinese, were included. After a rigorous screening process by 2 independent authors, with discrepancies resolved by a third, 10 eligible RCTs were selected for review. The results suggest that CDDP may provide significant therapeutic benefits, including anti-ischemic, vasodilatory, and antioxidative effects, with favorable safety profiles in both monotherapy and combination therapy. However, further multicenter RCTs are necessary to validate its long-term efficacy and safety. This review highlights the potential of CDDP as a complementary therapy for SAP and underscores the need for further evidence-based research to optimize its clinical use.

## 1. Introduction

Angina pectoris, a prevalent cardiovascular ailment, arises from myocardial ischemia triggered by inadequate coronary blood flow.^[1,2]^ Stable angina pectoris (SAP) is marked by predictable chest pain episodes, commonly induced by exertion or stress, which are alleviated by rest or the use of nitroglycerin. Although conventional treatments such as nitrates, β-blockers, and calcium channel blockers are widely used to manage SAP symptoms, they are often limited by concerns over long-term tolerability, adverse effects, and insufficient symptom control in certain patients.^[1,2]^ With demographic aging and shifting lifestyles, angina pectoris’s global incidence is on the rise, warranting urgent attention as a public health priority.^[3]^ Despite the effectiveness of current pharmacological therapies for SAP, they are often accompanied by significant limitations.^[4–6]^ Nitrates, while effective for acute angina relief, tend to lead to tolerance over time, which diminishes their long-term effectiveness. β-blockers and calcium channel blockers, although useful for symptom management and ischemia prevention, are prone to causing side effects such as bradycardia, fatigue, and hypotension. These side effects are particularly concerning in vulnerable populations, such as the elderly or patients with comorbidities like diabetes, asthma, or chronic obstructive pulmonary disease. Therefore, exploring novel therapeutic modalities is imperative in contemporary medical research.

Compound Danshen droplet pills (CDDP) is a traditional Chinese medicine formulation composed mainly of extracts from Salvia miltiorrhiza (*Danshen*), Panax notoginseng (*Sanqi*), and Borneol (*Bingpian*).^[7]^ The formulation has demonstrated a complex pharmacological profile, including anti-ischemic, vasodilatory, and antioxidative effects, which suggest its potential utility in managing conditions such as angina. Specifically, Salvia extract, which contains water-soluble phenolic acids like salvianolic acids, is known for its anti-ischemic and vasodilatory properties, while Panax notoginseng contributes saponins that may exert additional cardiovascular benefits.^[8]^

Emerging clinical evidence, combined with traditional medical principles, highlights CDDP’s potential in reducing angina recurrence without the risk of tolerance that is often associated with conventional therapies. Unlike some standard pharmacological treatments, which may lead to long-term tolerability issues or adverse effects, CDDP exhibits a favorable safety profile with minimal reported side effects. This distinction positions CDDP as an appealing alternative to conventional medications, especially considering the limitations of existing pharmacological interventions for SAP.

Current pharmacological treatments for SAP, while effective in symptom management, are often constrained by issues such as the development of drug tolerance, side effects, and limited long-term efficacy. These challenges underscore the need for alternative therapies, such as CDDP, which may offer a safer, more sustainable approach to angina management. The formulation of CDDP involves a specialized process that ensures a high dispersibility of its active ingredients, enhancing their bioavailability. This contributes to its robust pharmacological actions and supports its growing recognition in contemporary clinical settings as a complementary therapeutic strategy in cardiovascular care.^[7]^

Through a meticulous review of existing literature, this study intends to provide clinicians with evidence-based insights into the therapeutic potential of CDDP. Specifically, the analysis will focus on efficacy assessments, safety profiles, and elucidation of underlying mechanisms. By doing so, this study aims to facilitate informed decision-making among clinicians and offer novel therapeutic avenues for individuals afflicted with SAP.

## 2. Study search strategy and selection

### 2.1. Search strategy

This systematic review aimed to identify studies on the use of CDDP in the treatment of SAP. We conducted a comprehensive search using key terms such as “Compound Danshen Droplet Pills,” “CDDP,” and “Stable Angina Pectoris.” The databases searched included China National Knowledge Infrastructure and PubMed, covering publications from the inception of each database to July 31, 2024.

The search strategy was designed to capture relevant studies across multiple languages, particularly Chinese and English, to ensure the inclusion of both local and international research.

### 2.2. Inclusion criteria

Studies were eligible for inclusion if they satisfied the following criteria: the study specifically focused on evaluating the effects of CDDP as an intervention for SAP; the study employed a randomized controlled trial (RCT) design, as RCTs are considered the gold standard in clinical research for evaluating therapeutic efficacy and minimizing bias; and the study was published in peer-reviewed journals in either English or Chinese. These inclusion criteria were established to ensure that only high-quality evidence, directly relevant to the research question, was considered for analysis. By limiting inclusion to RCTs, the review aimed to ensure methodological rigor and avoid potential biases inherent in non-randomized study designs.

### 2.3. Exclusion criteria

Studies were excluded if they met any of the following conditions: the study investigated interventions that were unrelated to CDDP or focused on conditions other than SAP, as these would not provide relevant insights into the specific efficacy of CDDP for SAP; the study was a duplicate or had already been included in previous reviews, resulting in redundant data without contributing new information to the research question; the study employed non-randomized designs, such as cohort studies, case reports, case series, or systematic reviews, as these types of studies provide lower levels of evidence in comparison to randomized controlled trials and are more prone to bias; and the study was not published in a peer-reviewed journal, as this ensures the quality and validity of the research. These exclusion criteria were applied rigorously to maintain the integrity of the systematic review and ensure that only studies meeting the highest standards of evidence were included.

### 2.4. Study selection process

The initial selection process involved 2 independent authors screening the titles and abstracts of all identified studies according to the predetermined inclusion and exclusion criteria. Disagreements between the authors were resolved through discussion, and, when necessary, a third author was consulted to achieve consensus. This rigorous screening process ensured that only studies with the highest quality and relevance were included for further assessment.

**Figure 1.**
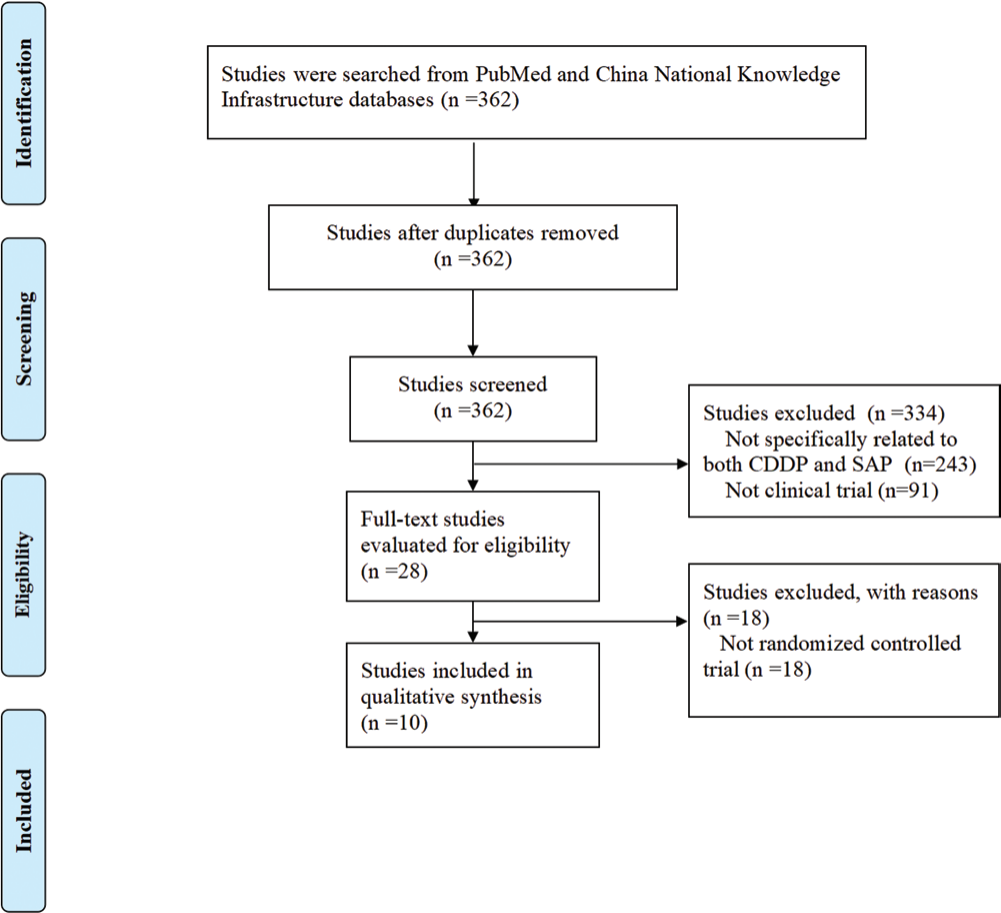
Flowchart of study selection.

A total of 362 studies were identified through the initial search. Following the application of the inclusion and exclusion criteria, 243 studies were excluded due to their lack of relevance to CDDP or SAP treatment (Fig. 1). The full texts of the remaining 28 studies were thoroughly reviewed for eligibility. Of these, 10 studies were deemed to meet the inclusion criteria and were ultimately included in the analysis. The remaining 18 studies were excluded because they did not meet the criteria for randomized controlled trials (Fig. 1).

## 3. Overview of angina pectoris

Angina pectoris is classified into stable and unstable forms based on symptom pattern and consistency. SAP exhibits relatively predictable symptoms triggered by exertion and alleviated by rest or nitroglycerin, while unstable angina involves more frequent and severe episodes, potentially signaling an impending acute coronary syndrome.

The pathophysiology of angina primarily stems from coronary hypoperfusion. Atherosclerosis, marked by lipid deposition and plaque formation within coronary arteries, is a primary causative factor, resulting in luminal narrowing and compromised blood flow to the myocardium. Additionally, thrombosis, coronary vasospasm, and endothelial dysfunction contribute to reduced coronary perfusion and provoke angina episodes.^[9–11]^ During these episodes, myocardial ischemia induces cellular hypoxia, leading to biochemical changes such as lactate accumulation, altered calcium handling, and membrane permeability alterations, ultimately manifesting as chest discomfort.^[9–11]^

Epidemiologically, SAP correlates closely with its pathophysiology.^[9,12]^ Its incidence rises with age, particularly among individuals aged 50 and older. Although men exhibit higher prevalence rates than women, this gender disparity diminishes post-menopause. Hypertension, dyslipidemia, diabetes, obesity, and other cardiovascular risk factors contribute significantly to SAP. Genetic predispositions may also influence disease susceptibility.^[13]^

In summary, a comprehensive comprehension of angina pectoris’s classification, etiology, and epidemiology is pivotal for accurate diagnosis, preventive measures, and therapeutic interventions. Future research endeavors should delve deeper into angina’s pathophysiological mechanisms, paving the way for enhanced understanding and the development of more efficacious treatment modalities to ameliorate patient outcomes and curtail cardiovascular morbidity and mortality.

## 4. Composition and pharmacological actions of CDDP

CDDP constitute a traditional Chinese medicine formulation comprising several medicinal herbs, notably Salvia, Panax notoginseng, and Borneol.^[7,8,14,15]^ These herbs are renowned in Traditional Chinese Medicine for their purported effects on promoting blood circulation, alleviating *Blood* stasis, relaxing tendons, activating collaterals, invigorating *Qi*, and nourishing *Blood.* Below is a detailed analysis of each key component of CDDP, elucidating their pharmacological actions within the cardiovascular system.^[7,8,14,15]^

Salvia (Salvia miltiorrhiza Bge.): Salvia is rich in bioactive compounds such as salvianolic acid and tanshinone, renowned for their antioxidative, anti-inflammatory, and antiplatelet aggregation properties.^[8,14,16]^ By inhibiting oxidative stress, mitigating endothelial injury, and enhancing myocardial blood flow, Salvia contributes to ameliorating microcirculatory dysfunction and alleviating symptoms associated with angina pectoris.^[8,14,16]^

Panax notoginseng: The primary bioactive constituents in Panax notoginseng, notably notoginsenosides, exhibit notable regulatory effects on cardiovascular function, anticoagulant properties, and coronary artery dilation.^[14,17,18]^ Through mechanisms such as enhanced nitric oxide synthesis, platelet aggregation inhibition, and myocardial cell injury reduction, Panax notoginseng aids in alleviating myocardial ischemia and reducing angina pectoris episodes.^[14,17,18]^

Borneol: Borneol exerts pharmacological effects such as heat-clearing, detoxification, blood circulation promotion, stasis resolution, pain relief, and muscle and tendon relaxation.^[14,19]^ It facilitates blood circulation, alleviates angina-related discomfort, and demonstrates vasodilatory properties, thus contributing to improved cardiovascular function.^[14,19,20]^

In summary, the diverse medicinal ingredients present in CDDP exert multifaceted beneficial effects on the cardiovascular system, encompassing microcirculatory enhancement, vasodilation, antioxidation, and anti-inflammation. These synergistic pharmacological actions collectively offer a safe and efficacious therapeutic option for individuals suffering from angina pectoris.

## 5. Clinical research progress of CDDP in treating SAP

Clinical studies on the treatment of SAP with CDDP involve 2 main aspects: single therapy research and combination therapy research^[21–27]^ (Tables 1 and 2). Below is a review of the clinical research progress.

**Table 1:** Clinical studies of CDDP for patients with SAP

References	Patients	Study type	Treatment	Main results	Main findings	AEs
Wang et al 2021^[21]^	Patients with SAP	RCT	CDDP	The treatment group had a higher effective rate (91.49%) than the control (74.47%) (*P* < .05). Both groups showed improved angina, LVEF, LVESD, viscosity, and quality of life, with better results in the treatment group (*P* < .05).	Reducing the frequency and duration of angina attacks while improving blood rheology, cardiac function, and quality of life	NR
Li et al 2020^[22]^	Patients with SAP	RCT	CDDP	The treatment group had fewer angina attacks, shorter duration, lower symptom scores, and a higher effective rate than the control group (*P* < .05). Serum ET-1 and hs-CRP levels were lower, and NO was higher (*P* < .05). Adverse reactions were similar (*P *> .05).	Improved SAP symptoms in patients with Qi stagnation and blood stasis, and lowered ET-1 and hs-CRP levels while raising nitric oxide levels	The treatment group had 2 cases of pain, 2 of nausea/vomiting, 1 of diarrhea, and 1 of dizziness. The control group had 1 case of discomfort, 1 of nausea/vomiting, 2 of diarrhea, and 1 of headache.
Zhou 2019^[23]^	Patients with SAP	RCT	CDDP	The treatment group showed superior efficacy, with a 50% reduction in angina frequency (*P* < .05). No significant differences were found in angina attacks or cardiac function (*P* > .05), though the treatment group had better results (*P* < .05).	Clinically effective and improves heart function	No adverse reactions occurred.
Zhang 2018^[24]^	Patients with SAP	RCT	CDDP	After treatment, the observation group showed a significantly greater reduction in whole blood viscosity, hematocrit, and fibrinogen compared to the control group (*P* < .05).	Effectively improved symptoms of SAP	NR
Wang et al 2017^[25]^	Patients with SAP	RCT	CDDP	After treatment, the treatment group showed greater improvements in angina frequency, blood lipid profiles (lower TC, LDL, higher HDL), and reduced CRP and BNP levels compared to the control group (*P* < .05), with a higher total effective rate (*P* < .05).	Reduces CRP and brain natriuretic peptide levels may work by regulating biofactors to relieve SAP symptoms	The treatment group had 3 cases of gastrointestinal reactions, while the control group had 5 cases, including 2 patients with orthostatic hypotension.
Zhao 2016^[26]^	Patients with SAP	RCT	CDDP	The treatment group had higher treatment efficacy (94.59%) and electrocardiogram effectiveness (86.49%) compared to the control group (75.67%, 64.86%, *P* < .05).	Clinical and electrocardiogram total effective rates have significantly improved	NR
Zhang et al 2016^[27]^	Patients with SAP	RCT	CDDP	The treatment group had a significantly higher total effective rate than the control group (*P* < .05).	Clinical total effective rate has significantly improved	NR

AEs = adverse effects, CDDP = compound Danshen dripping pills, ET-1 = serum endothelin-1, HDL = high-density lipoprotein, hs-CRP = high sensitivity C-reactive protein, LDL = low-density lipoprotein, LVEF = left ventricular ejection fraction, LVESD = left ventricular end-systolic diameter, NO = nitric oxide, NR = not reported, RCT = randomized controlled trial, SAP = stable angina pectoris, TC = cholesterol.

**Table 2:** Clinical studies of CDDP combined IDT for patients with SAP

References	Patients	Study type	Treatment	Main results	Main findings	AEs
Jiang et al 2011^[28]^	Patients with SAP	RCT	CDDP + IDT	The treatment group had 67.5% anti-angina efficacy, 62.5% ECG improvement, and reduced TC by 21.6% and TG by 18.3%. The control group showed lower efficacy and smaller reductions in TC and TG.	Combination of CDDP and IDT in treating coronary heart disease-related SAP can dilate coronary arteries, improve myocardial blood flow, reduce the dosage of IDT, and lower the risk of tolerance, while providing quick clinical efficacy with minimal adverse effects	In the treatment group, 2 patients had mild stomach discomfort. In the control group, 2 patients discontinued due to severe headache and nausea.
Cao 2011^[29]^	Patients with SAP	RCT	CDDP + IDT	The treatment group showed a 93% total efficacy rate, significantly higher than the 62.5% in the control group (*P *< .01). For ECG improvement, the treatment group had an 81.4% efficacy, compared to 50% in the control group (*P* < .01).	Combination of CDDP and IDT is more effective in treating SAP than using IDT alone	In the treatment group, 2 patients had stomach discomfort, relieved with ranitidine. In the control group, 4 had flushing and dizziness, and 2 had headaches, but treatment continued.
Jiang et al 2009^[30]^	Patients with SAP	RCT	CDDP + IDT	The treatment group had 76.0% symptom improvement, higher than 52.0% in the control (*P* < .05). Treatment group was more effective than the control (*P* < .05). ECG improvement was 67% vs. 42% (*P* < .05)	Combination of CDDP and IDT is more effective in treating SAP than using IDT alone	No significant AEs occurred in the treatment group. In the control group, 4 cases of headache, 2 discontinued treatment, and 3 cases of flushing/dizziness resolved without stopping the medication.

Es = adverse effects, CDDP = compound Danshen dripping pills, ECG = electrocardiogram, IDT = isosorbide dinitrate tablets, RCT = randomized controlled trial, SAP = stable angina pectoris, TC = cholesterol, TG = triglycerides.

### 5.1. Single therapy research

Early clinical studies showed promising results for using CDDP to treat SAP. These studies highlighted a significant reduction in myocardial ischemic symptoms, such as decreased pain intensity, while indicating that CDDP was generally well-tolerated by patients. Subsequent randomized controlled trials (RCTs) supported these findings by showing that patients receiving CDDP experienced a notable reduction in angina symptoms compared to control managements^[21–27]^ (Table 1). The RCTs also suggested that CDDP might reduce myocardial ischemia and improve the heart’s tolerance to ischemic episodes. CDDP’s mechanisms in reducing angina symptoms appear to involve improving blood rheology (the study of blood flow properties, including viscosity and elasticity) and cardiac function. Some studies also noted that CDDP could influence biochemical markers, such as reducing high-sensitivity C-reactive protein and endothelin-1 while increasing nitric oxide (NO) levels.^[22]^ Long-term observational studies have provided additional evidence of CDDP’s effectiveness in reducing the recurrence rate of angina attacks. These studies also indicated that prolonged use of CDDP might have a positive impact on overall cardiovascular function.

Overall, CDDP has demonstrated significant clinical benefits for SAP patients. Its consistent efficacy across multiple studies suggests that it could be a valuable addition to treatment protocols for SAP. The potential to improve the quality of life for patients and reduce the risk of recurrent angina makes it a noteworthy therapy option^[21–27]^ (Table 1).

### 5.2. Combination therapy research

The studies examined the impact of combining CDDP with other pharmacotherapies, specifically in patients with SAP. Participants in these studies were diagnosed with SAP and varied in terms of age, gender, and underlying health conditions^[28–30]^ (Table 2). The combination therapy typically involved CDDP and additional cardiovascular medications, such as Isosorbide dinitrate tablets (IDT). These combinations aimed to assess the synergistic effects of combining traditional Chinese medicine with conventional pharmacological treatments^[28–30]^ (Table 2).

Clinical trials reported that the combination of CDDP and IDT produced significant improvements in SAP symptoms, with participants experiencing reduced frequency and severity of angina attacks. The studies suggested that this combination therapy could improve myocardial blood flow, dilate coronary arteries, and reduce the dosage of IDT required for effective treatment.^[28–30]^ By combining CDDP with IDT, studies found a reduced risk of developing tolerance to IDT, a common issue in long-term nitrate therapy. Additionally, the adverse effects of the combination therapy were minimal, indicating a favorable safety profile.^[28]^ The studies indicated that combining CDDP with other cardiovascular medications might have a positive impact on long-term patient outcomes, potentially reducing the risk of future cardiovascular events and enhancing overall heart function.^[28–30]^

Overall, the combination of CDDP and IDT, among other pharmacotherapies, has shown promise in treating SAP. The observed benefits in terms of efficacy, safety, and reduced tolerance suggest that this combination therapy could be a valuable addition to existing treatment protocols for SAP^[28–30]^ (Table 2).

### 5.3. Efficacy and safety evaluation

A systematic appraisal of CDDP’s efficacy and safety in SAP treatment is imperative.^[21–30]^ Evaluation of efficacy encompasses comprehensive assessments of angina symptom improvement, myocardial ischemia mitigation, and enhancements in patients’ overall quality of life. Safety scrutiny encompasses rigorous monitoring of adverse reaction incidences and potential drug interactions.^[31,32]^ Findings from multiple clinical trials consistently affirm the significant efficacy of CDDP in managing SAP, coupled with favorable safety profiles characterized by minimal adverse reactions and high patient tolerability^[21–32]^ (Table 1 and 2).

#### 5.3.1. Efficacy of CDDP in SAP Treatment

Numerous studies have consistently demonstrated the positive effects of CDDP in managing SAP^[21–32]^ (Tables 1 and 2). Clinical trials indicate significant improvements in angina symptoms and reductions in the frequency and severity of myocardial ischemia.^[21–32]^ Additionally, patient quality of life is enhanced following CDDP therapy, making it a potentially valuable therapeutic option for SAP management. These results are summarized in Table 1, which provides a comparative overview of the efficacy outcomes across various clinical trials.

#### 5.3.2. Safety profile of CDDP

In terms of safety, findings from multiple clinical studies highlight that CDDP is generally well-tolerated by patients^[21–32]^ (Tables 1 and 2). Adverse reactions, when they occur, are typically mild to moderate in severity.^[22,25,28–30]^ Commonly reported side effects include nausea, dizziness, and fatigue; however, these are infrequent and generally transient. Table 2 consolidates the safety data from several trials, offering a detailed breakdown of adverse reactions, their incidence rates, and the overall impact on patient adherence to treatment.

While the incidence of severe adverse events is low, the safety profile of CDDP necessitates close monitoring, particularly in patients with preexisting conditions or those on concomitant medications.^[22,25,28–30]^ The drug’s favorable tolerability and minimal side effects contribute to its clinical viability and patient retention rates, further supporting its potential as a therapeutic agent in SAP management.

In summary, CDDP presents significant promise as a therapeutic agent for SAP, either as a monotherapy or in combination with other treatments. Its efficacy in improving angina symptoms and mitigating myocardial ischemia is well-documented, while its safety profile remains favorable, with minimal adverse effects and high patient tolerability. Nonetheless, to substantiate these findings, further large-scale, multicenter clinical trials are essential. Such studies will help confirm CDDP’s long-term efficacy, refine its safety parameters, elucidate its mechanisms of action, and define its optimal therapeutic role in the management of angina.

## 6. Mechanism of CDDP in the treatment of angina

### 6.1. Antioxidant action

Salvia, a key component of CDDP, contains bioactive compounds such as tanshinones and salvianolic acids, which demonstrate robust antioxidant properties.^[33]^ These compounds mitigate oxidative stress by scavenging free radicals, thereby protecting cardiovascular cells and tissues.^[33]^ By inhibiting oxidative stress, CDDP contribute to alleviating the severity of angina.

### 6.2. Vasodilatory action

Panax notoginseng, another component of CDDP, harbors active constituents like notoginsenosides, which facilitate the release of NO, leading to vascular smooth muscle relaxation and subsequent vasodilation.^[34]^ This mechanism enhances coronary and peripheral blood flow, thus mitigating the frequency and intensity of angina attacks.^[34]^

### 6.3. Anti-inflammatory action

CDDP exhibit anti-inflammatory properties. Considering the association between inflammation and angina progression, these components suppress inflammatory mediator release and mitigate inflammatory responses, ameliorating angina severity and frequency.^[8,35–37]^

### 6.4. Other mechanisms

Apart from the aforementioned mechanisms, CDDP may modulate lipid metabolism, enhance myocardial metabolism, and augment myocardial contractility.^[38–41]^ Additionally, they could exert analgesic and anxiolytic effects, further alleviating pain and anxiety in angina patients.

In summary, the multifaceted mechanism of CDDP in angina treatment encompasses antioxidant, vasodilatory, anti-inflammatory, and potentially other pharmacological actions. These mechanisms synergistically contribute to a comprehensive therapeutic approach for angina management.

## 7. Clinical application prospects and outlook of CDDP in the treatment of SAP

### 7.1. Advantages and limitations

#### 7.1.1. Advantages

CDDP, rooted in traditional Chinese medicine, feature a rich composition including Salvia, Panax notoginseng, and Borneol.^[7,8]^ These components are believed to exert synergistic effects, enhancing cardiovascular function and alleviating angina symptoms according to traditional Chinese medicine principles.^[28–30]^ Long-standing clinical use has demonstrated relatively high safety and minimal adverse reactions associated with CDDP. Their suitability for long-term administration and convenient oral intake contribute to improved patient compliance.

#### 7.1.2. Limitations

The therapeutic efficacy of CDDP tends to manifest gradually and necessitates prolonged administration for significant outcomes, making them less suitable for acute angina episodes.^[7,8]^ Despite their extensive use, the precise pharmacological mechanisms underlying CDDP remain incompletely understood, warranting further clinical and basic research to elucidate their mode of action. Furthermore, variability in factors such as raw material quality, formulation ratios, and manufacturing processes may lead to batch-to-batch inconsistencies in efficacy, necessitating rigorous quality control measures and standardized production protocols. Additionally, patient adherence to long-term treatment regimens may vary, potentially affecting the consistency of clinical outcomes. Therefore, the impact of patient compliance on treatment efficacy warrants further investigation. Another limitation is the generalizability of the findings. The sample size and patient demographics in the current studies may limit the applicability of the results to broader populations. Larger, more diverse cohorts are needed to better assess the broader effectiveness and applicability of CDDP treatment. Future studies should aim to include more diverse patient populations to strengthen the external validity of the findings.

### 7.2. Future research directions

#### 7.2.1. Clinical research

Rigorous clinical studies are required to validate the efficacy and safety of CDDP across diverse angina patient populations. These studies should investigate optimal dosing regimens and treatment protocols.

#### 7.2.2. Pharmacological studies

In-depth pharmacological investigations are essential to uncover the precise mechanisms through which CDDP exert their therapeutic effects on the cardiovascular system. Molecular and cellular studies can provide insights into their actions.

#### 7.2.3. Integration of modern techniques

Integration of modern approaches such as bioinformatics and pharmacokinetics can augment traditional research methods, offering a comprehensive understanding of CDDP’s pharmacodynamics and pharmacokinetics.

#### 7.2.4. Quality control and standardization

Emphasis should be placed on enhancing quality control measures and standardizing production processes to ensure consistency and reliability in the formulation of CDDP. This will contribute to their efficacy and safety in clinical practice.

## 8. Summary

The CDDP, a traditional Chinese medicine formulation, exhibits promising potential in managing SAP. This paper provides a comprehensive review of its therapeutic efficacy, pharmacological mechanisms, and future research directions. Delving into the therapeutic efficacy, this review critically evaluates the clinical outcomes and safety profile of the CDDP, both as a standalone treatment and in combination with other medications. Additionally, it elucidates the underlying pharmacological mechanisms responsible for its therapeutic effects in alleviating SAP symptoms. Despite significant advancements, unresolved challenges persist in the clinical application of the CDDP for SAP treatment. This section highlights the need for further investigation into its pharmacokinetics, optimal dosage regimens, and long-term safety profile through robust clinical trials.

In summary, the CDDP emerges as a promising therapeutic option for SAP. Through a meticulous review of its therapeutic efficacy, pharmacological mechanisms, and research challenges, this paper underscores its potential significance in clinical practice and emphasizes the need for continued scientific inquiry to optimize its use.

## Author contributions

**Conceptualization:** Xiao-feng Liu, Xiao-xiao Zhu.

**Data curation:** Xiao-feng Liu, Xiao-xiao Zhu.

**Investigation:** Xiao-xiao Zhu.

**Methodology:** Xiao-feng Liu, Xiao-xiao Zhu.

**Project administration:** Xiao-xiao Zhu.

**Resources:** Xiao-feng Liu.

**Supervision:** Xiao-xiao Zhu.

**Validation:** Xiao-feng Liu, Xiao-xiao Zhu.

**Visualization:** Xiao-feng Liu, Xiao-xiao Zhu.

**Writing – original draft:** Xiao-feng Liu, Xiao-xiao Zhu.

**Writing – review & editing:** Xiao-feng Liu, Xiao-xiao Zhu.
